# Mapping knowledge structure and themes trends of cancer-associated fibroblasts: a text-mining study

**DOI:** 10.3389/fmolb.2023.1302016

**Published:** 2023-12-04

**Authors:** Kunming Cheng, Wanqing Li, Haiyang Wu, Cheng Li

**Affiliations:** ^1^ Department of Intensive Care Unit, The Second Affiliated Hospital of Zhengzhou University, Zhengzhou, China; ^2^ Department of Operating Room, Xiangyang Central Hospital, Affiliated Hospital of Hubei University of Arts and Science, Xiangyang, China; ^3^ Department of Clinical College of Neurology, Neurosurgery and Neurorehabilitation, Tianjin Medical University, Tianjin, China; ^4^ Duke Molecular Physiology Institute, Duke University School of Medicine, Durham, NC, United States; ^5^ Department of Orthopaedic Surgery, Beijing Jishuitan Hospital, Capital Medical University, Beijing, China; ^6^ Center for Musculoskeletal Surgery (CMSC), Charité-Universitätsmedizin Berlin, Corporate Member of Freie Universität Berlin, Humboldt University of Berlin, Berlin Institute of Health, Berlin, Germany

**Keywords:** bibliometrics, hotspots, cancer-associated fibroblasts, citespace, VOSviewer

## Abstract

**Introduction:** Cancer-associated fibroblasts (CAFs) constitute an important component of the tumor microenvironment, participating in various facets of cancer advancement and being recognized as contributors to tumor immune evasion. The role of CAFs in various tumor types has attracted increasing attention recently. In this work, we conducted a comprehensive bibliometric analysis to uncover research trajectories and highlight emerging areas in the field of CAFs.

**Methods:** A systematic search was performed within the Web of Science Core Collection to identify articles/reviews on CAFs published between 2000 and 2023. Leveraging advanced bibliometric tools such as VOSviewer, CiteSpace, and online website, we examined and visualized publication trends, geographic contributions, institutional affiliations, journal prominence, author collaborations, and noteworthy references, keywords, and genes.

**Results:** Our analysis included 5,190 publications, indicating a rapid growth trend in both annual publications and citations related to CAFs. China and the United States emerged as the foremost contributors in terms of publications, funding agencies, and international collaborations. Breast cancer was the most studied tumor, followed by colorectal cancer, pancreatic cancer, prostate cancer, and gastric cancer. Based on co-occurrence and bursting keywords, we identified the following research topics including immune cells (T cells, B-cells, tumor-associated macrophages), tumor immune microenvironment (antitumor immunity, immune infiltration, immunosuppression), immunotherapy (PD-L1), microRNAs (miRNA), extracellular vesicles (exosome), multiple tumors (pancreatic ductal adenocarcinoma, bladder cancer, head and neck squamous cell carcinoma), antitumor agents (gemcitabine, cisplatin resistance), bioinformatics (pan-cancer), epithelial-mesenchymal transition (stemness), FAPI PET/CT, DNA methylation, etc., may receive sustained attention in the future. Furthermore, TGFB1, IL-6, TNF, TP53, and VEGFA emerged as the top 5 genes that have garnered the greatest research attention in the field of CAFs. The KEGG enrichment analysis highlighted that the top 20 most studied genes were mainly associated with HIF-1 and Toll-like receptor signaling pathways.

**Discussion:** In sum, our bibliometric analysis offers a comprehensive overview of the research landscape in the field of CAFs. It encompasses the current state, evolving patterns, and prospective avenues of exploration, with special attention to the potential advancements in tumor immune microenvironment.

## Introduction

As is known to all, the initiation of tumorigenesis involves multiple biological mechanisms and complex factors. The tumor microenvironment (TME) constitutes an exceptionally intricate internal milieu, which comprises cancer cells, tumor stromal cells, cellular metabolic byproducts, and a variety of physicochemical factors such as pH and hypoxia (Kessenbrock et al., 2010). These components play a pivotal role in modulating the biological behavior of tumors. Among them, Cancer-associated fibroblasts (CAFs), also known as activated fibroblasts exhibiting markers of myofibroblasts, stand out as one of the most abundant stromal cell types in tumor stroma (Park et al., 2020). At present, it is generally considered that CAFs primarily originate from inherent fibroblasts or stellate cells within the tissue, which undergo transformation into CAFs due to stimuli from tumor-derived factors like Transforming Growth Factor-beta (TGF-β), platelet derived growth factor (PDGF), fibroblast growth factor 2 (FGF-2), and so on (Park et al., 2020; Fang et al., 2023). Furthermore, epithelial cells, endothelial cells, and bone marrow-derived stem cells (BMSCs) within the tumor tissue could also differentiate into CAFs. A considerable amount of research has indicated that CAFs play an essential role in regulating tumor initiation, growth, metastasis, and drug resistance (Eckert et al., 2019; Zhang et al., 2023a; Huang et al., 2023). For example, CAFs secrete a multitude of cytokines and metabolic products, engaging in crosstalk with tumor cells and other stromal cells, thereby suppressing immune cell function and promoting tumor progression (Guo et al., 2023). In recent years, it has also been revealed that CAFs play a role in antigen cross-presentation. Through *Fas/Fas-*ligand system-mediated pathway, CD8^+^ T lymphocytes are eliminated, consequently enabling tumor cells to evade immune system surveillance and escape immune attacks (Lakins et al., 2018). In addition, CAFs remodel the extracellular matrix structure, creating a barrier that impedes the infiltration of drugs and immune cells, thereby restraining the infiltration of immune cells recruited by tumor-secreted chemokines and the penetration of anti-tumor drugs (Mao et al., 2021).

Due to their ease of isolation and inherent plasticity, CAFs have been extensively studied in recent years. CAFs are being considered as promising targets for the development of novel anticancer therapies. Targeted modulation of CAFs or overcoming their barrier function to inhibit tumor progression and overcome drug resistance represents a novel approach to enhancing the efficacy of cancer treatment (Mao et al., 2021). Nevertheless, owing to the lack of specific markers, ongoing research into CAFs, including their origin, phenotypic variations, and functionalities, encounters many challenges (Öhlund et al., 2017; Xu et al., 2023). Meanwhile, most of the current research on CAFs are based on cellular level or animal models, clinical trials directly targeting CAFs remain limited (Mu et al., 2021). And to our knowledge, there is still no effective strategy that has translated into a successful clinical outcome. In view of this, there has been increasing research interest in CAFs recently and a great number of related studies have been published as the research goes on. However, substantial growth of data has resulted in information overload, making it increasingly challenging to fully leverage existing knowledge and literature. Researchers may find it is difficult to comprehend the latest research trends in the field from multidimensional perspectives.

Bibliometrics, first proposed in 1969 by Alan Richard, is an important tool that applies mathematical and statistical methods to aid researchers in effortlessly, quantitatively, and qualitatively grasping the research trend in a specific research field (Li et al., 2022; Cheng et al., 2023a). In recent years, with the development of open-source bibliometric software, bibliometric analyses have been frequently adopted in the biomedical domain (Synnestvedt et al., 2005; van Eck and Waltman, 2010). Take TME as an example, several bibliometric studies have thoroughly explored the global publication trends and emerging focus on tumor immune microenvironment (Wang et al., 2022; Liu et al., 2023), tumor-associated macrophage (Zhou et al., 2023), inflammatory tumor microenvironment (Zhang et al., 2022), hematological tumor microenvironment (Chen et al., 2021), and so on. However, to the best of our knowledge, no studies have adopted a bibliometric perspective to analyze the current research trend and hotspots in the field of CAFs. To fill this gap, we employed three bibliometric tools that are recognized as authoritative within the field of bibliometrics to conduct an exhaustive analysis centered on publications related to CAFs from 2000 onwards.

## Materials and methods

### Data source

The Web of Science (WoS) of Clarivate Analytics comprises an extensive collection of academic journals and peer-reviewed articles, widely recognized as one of the most authoritative and comprehensive public databases across multiple disciplines (Song et al., 2022; Zhou et al., 2023). The data for this bibliometric analysis were sourced from the Science Citation Index Expanded (SCIE) in WoS Core Collection (WoSCC) database, which is considered the optimal data source to perform bibliometric studies.

### Data retrieval strategy

**FIGURE 1:**
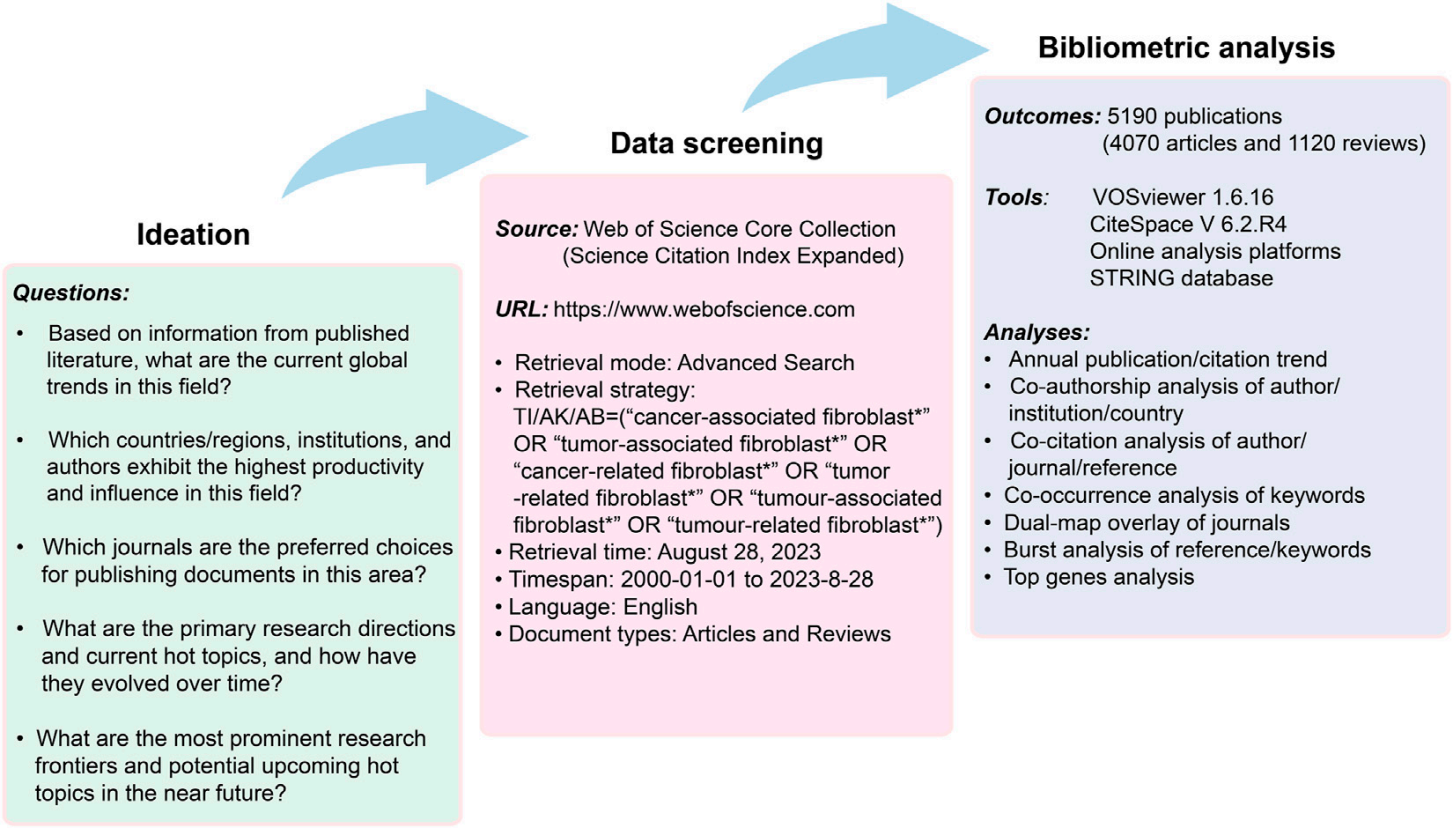
Detailed flowchart of literature screening and data extraction.

After discussions with several experts in the field, and with reference to the medical subject headings (MeSH), the search topic for our study focused on the terms: “cancer-associated fibroblast*,” “tumor-associated fibroblast*,” “cancer-related fibroblast*,” “tumor-related fibroblast*,” “tumour-associated fibroblast*,” “tumour-related fibroblast*.” The wildcards * represent root word truncation of one or more other characters. For example, fibroblast* could refers to fibroblast or fibroblasts. The field tags used were TI (article titles), AK (author keywords), and AB (article abstracts). The retrieval date was from 1 January 2020 to 28 August 2023. Document types were restricted to “research article” and “review”, and the language was restricted to “English.” The detailed flowchart of literature screening and data extraction was shown in Figure 1.

### Data collection

Afterwards, all literature records meeting the above criteria were acquired and exported in the plain text file or tab delimiter file format, encompassing a “full record and cited references” content approach. This content included details such as titles, keywords, citation counts, publication dates, countries/regions, researchers, affiliations, academic journals, impact factor (IF), H-index, funding agencies, and other related indicators. These records were then utilized for subsequent visualization and bibliometric analysis.

### Data analysis

In this investigation, the primary statistical methodology employed was descriptive analysis. Microsoft Excel 2019 was employed to conduct curve fitting on the annual publication and citation counts. The selection of the optimal fitting model was guided by the attainment of the highest determination coefficient (*R* ^2^). For determining the annual growth rate of publications over time, the following dedicated formula was utilized: annual growth rate = [(number of papers in the last year ÷ number of publications in the first year)^1/(last year - first year)^—1] × 100 (Wu et al., 2021). Additionally, Pearson’s correlation coefficient test was utilized to evaluate the correlation between citations and publications. A *p*-value below 0.05 was considered indicative of statistical significance.

Bibliometric visualization was conducted using CiteSpace 6.2 R4, VOSviewer 1.6.16, and one online analytical platform [https://bibliometric.com/)]. Of them, VOSviewer was used to conduct paper citation analysis, countries and authors co-authorship analysis, journals co-citation analysis, as well as co-occurrence analysis of keywords (van Eck and Waltman, 2010). The specific parameters of VOSviewer software accepted the default settings itself. This software could construct three different visual maps including the network, density and overlay visualization maps. Generally speaking, different maps with various nodes and lines represent different meanings, which have been described in detail in the figure legends. In addition, detailed explanation of these maps can be found in the figure legends. Moreover, the user manual of this software is available at https://www.vosviewer.com/documentation/Manual_VOSviewer_1.6.16.pdf. CiteSpace was used to perform dual-map overlay of journals and bursts analysis of keywords/references (Synnestvedt et al., 2005). The parameters of CiteSpace were set as follows: Method (LLR), time slicing (2000–2023), years per slice (1/2), term source (all selection), node type (choose one at a time), and selection criteria (top 30/50 objects). Also, a manual about CiteSpace software is available at http://cluster.ischool.drexel.edu/∼cchen/citespace/CiteSpaceManual.pdf. And the online platform was used to investigate collaboration networks among countries/regions. In addition, we conducted an analysis centered on genes associated with CAFs. To facilitate this, we utilized the online resource accessible at https://www.citexs.com/Summary. This platform allowed us to aggregate gene data from various studies within the designated field, with the literature sourced from the PubMed database. Furthermore, the protein-protein interaction network and functional enrichment analysis of the most extensively studied genes were carried out using the Search Tool for the Retrieval of Interacting Genes (STRING) database, which is accessible at https://string-db.org/(Yang et al., 2023).

## Results

### Publication outputs and citation trends on a global scale

**FIGURE 2:**
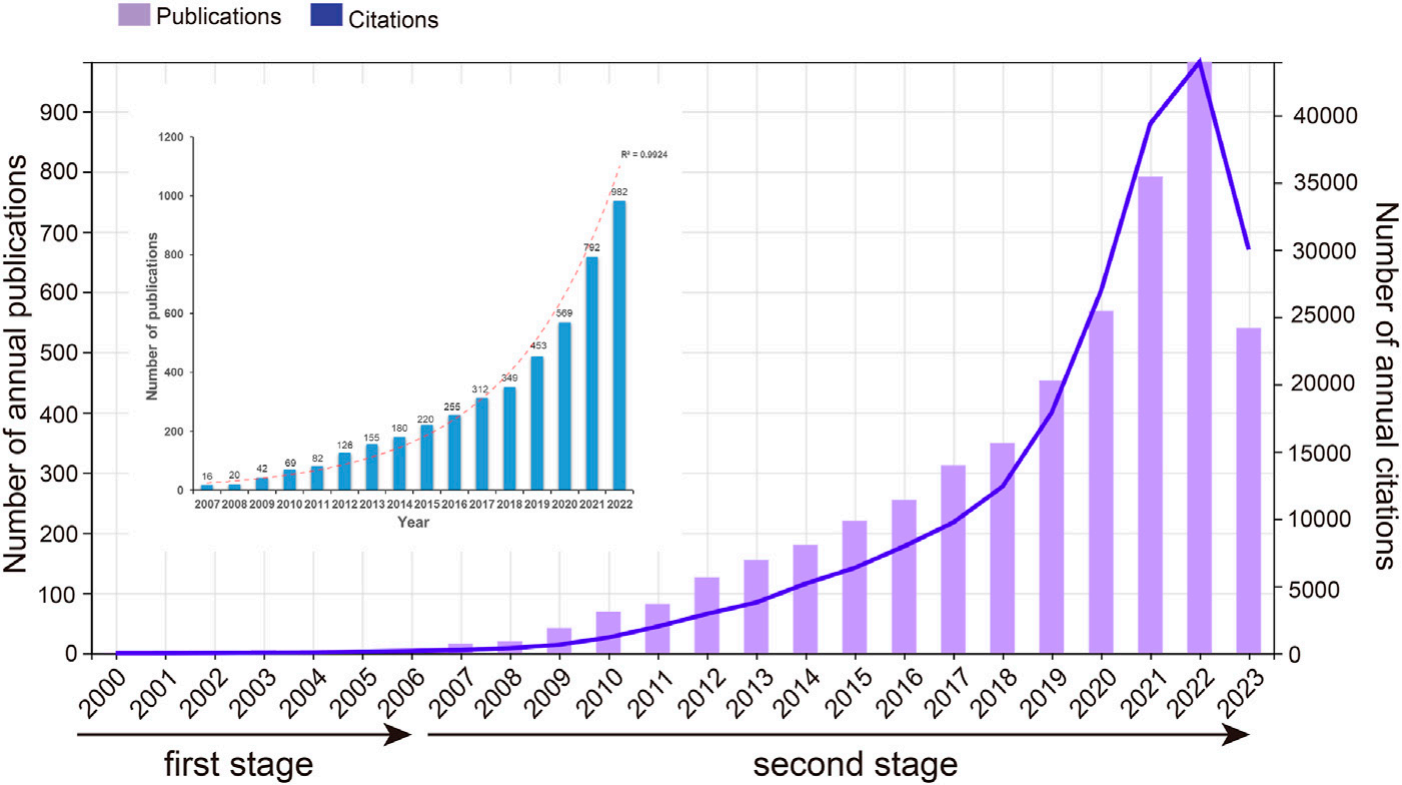
Number of annual publications and citations in CAFs.

In accordance with the literature search and screening methodology depicted in Figure 1, a total of 5,190 documents were ultimately incorporated. This encompassed 4,070 original articles and 1,120 reviews spanning the years 2000–2023. The cumulative citations for all these publications amounted to 211232 instances, with an average of approximately 40.7 citations per document. The H-index for the entire selection of CAFs-related documents was computed at 189, indicating that a minimum of 189 articles from this selection have received at least 189 citations each. As shown in Figure 2, the publication output experienced a notable surge, starting from 1 in 2000 and peaking at 982 in 2022. The mean growth rate for these publications from 2000 to 2022 was calculated to be 36.8%. Furthermore, we divided the entire CAFs research process into two distinct periods: the initial stage (2000–2006) and the rapid development stage (2007–2023). The *R* ^2^ for annual publication numbers from 2007 to 2022 was determined to be 0.9924, demonstrating a robust fit of the exponential growth model. Likewise, the annual citation numbers exhibited a parallel upward trajectory, in harmony with the escalating publication output. Additionally, a statistically significant correlation (*r* = 0.989, *p* < 0.001) was observed between publications and citations.

### Visual analysis of active journals and research areas

**FIGURE 3:**
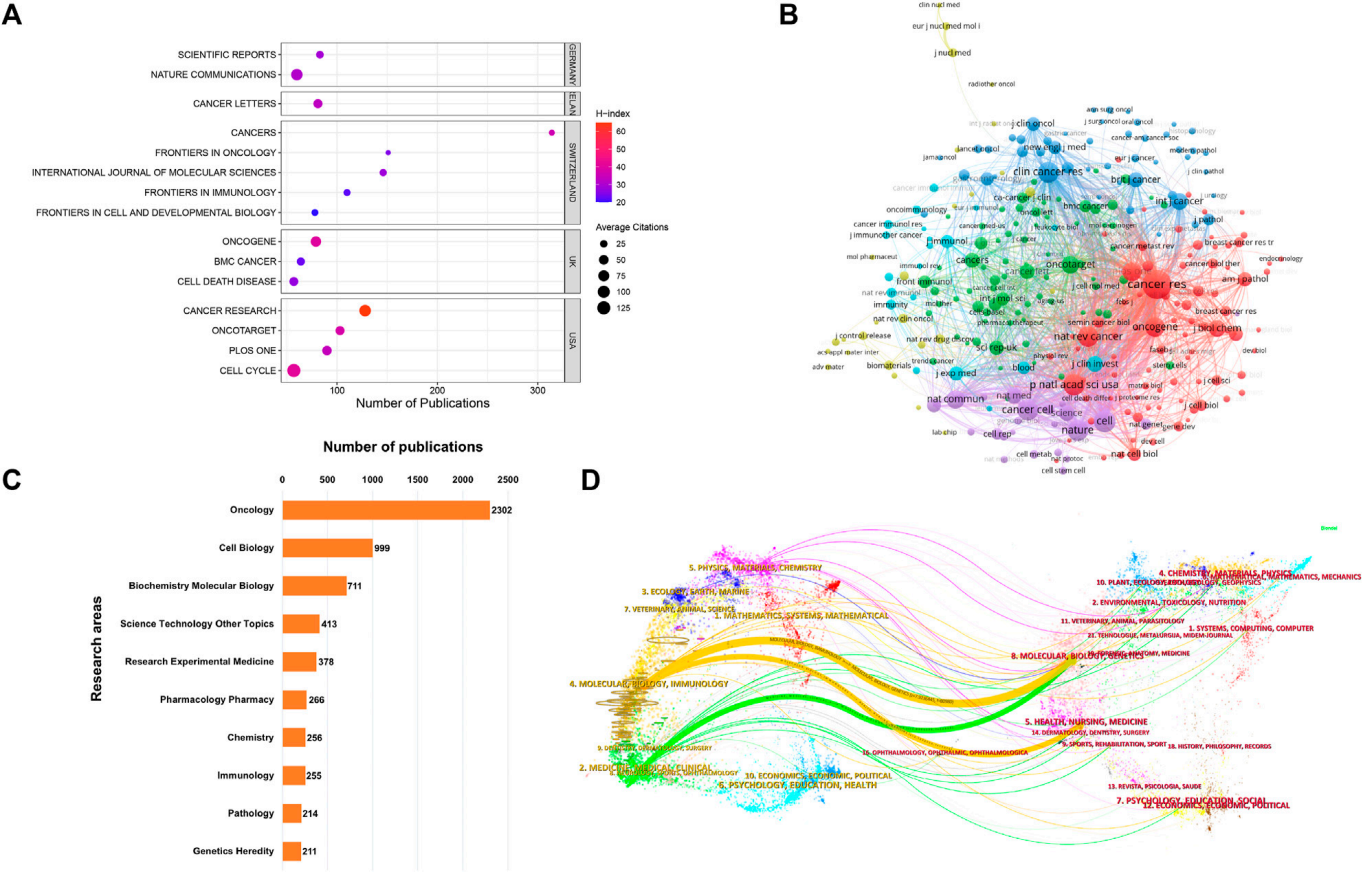
**(A)** Dot plot of the top 15 journals with the most publications. **(B)** Network visualization map depicting 277 co-cited journals with over 200 citations each. The size of the nodes reflects the number of citations accumulated by these journals. **(C)** Top 10 research areas for CAFs. **(D)** Dual-map overlay illustrating journals in the field of CAFs. Each point on the map corresponds to a specific journal. The left side of the map shows the citing journals, while the right side features the cited journals.

Examining the distribution of studies across various journals could provide valuable insights to individuals within the academic publishing realm. This analysis could help them make more informed decisions when selecting publication channels that best match their research content and intended audience. Figure 3A presents a dot plot of the top 15 journals with the most publications. Among these, *Cancers* (*n* = 314) held the highest publication count, followed closely by *Frontiers in Oncology* (*n* = 151) and *International Journal of Molecular Sciences* (*n* = 146). These journals predominantly fell within the Q1/Q2 categories, according to the recent Journal Citation Report. *Nature Communications* boasted the highest IF at 16.6, while the *Cancer Research* led with the highest H-index at 65. Figure 3B provides a network visualization map, depicting 277 co-cited journals with over 200 citations each. The top three journals with the most significant Total Link Strength (TLS) were *Cancer Research*, *Cell*, and *PNAS*. Additionally, the WoSCC database could categorize these publications into various research areas based on their subject categories. Figure 3C displays the top 10 research domains, organized by their publication counts. The research topic oncology contributes 44.4% publications, followed by cell biology which account for 19.2%. Figure 3D exhibits a dual-map overlay of journals, revealing the positioning of studies associated with CAFs within the broader spectrum of academic disciplines. Each data point on this map corresponds to a specific journal. The left side of the map illustrates the citing relationships, while the right side portrays the journals being cited. The connecting curve between them represents the flow of citations. This dual-map overlay provides a comprehensive visualization of the citation context. It showed that publications in the MOLECULAR, BIOLOGY, and IMMUNOLOGY fields are mainly influenced by publications in the MOLECULAR, BIOLOGY, and GENETICS field and HEALTH, NURSING, and MEDICINE fields (orange trajectory). Moreover, articles published in the MEDICINE, MEDICAL, and CLINICAL fields are mainly influenced by publications in the fields of MOLECULAR, BIOLOGY, and GENETICS (green trajectory).

### Visual analysis of principal contributors and collaborative network

#### Contributions of countries/regions and supported funds

**FIGURE 4:**
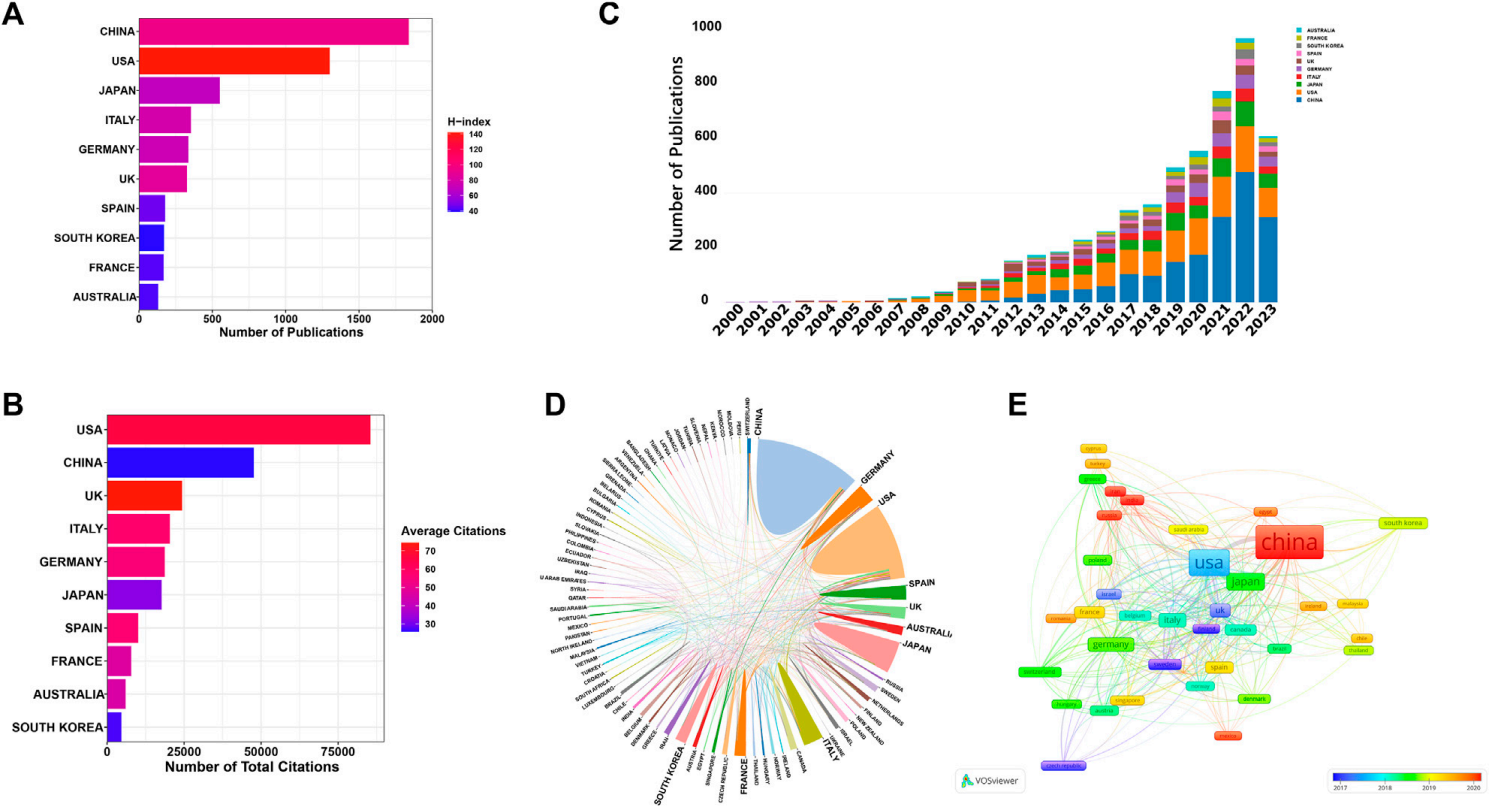
**(A)** The leading 10 contributors based on their publication outputs. **(B)** The leading 10 contributors based on their citations. **(C)** The annual publication trends of the top 10 contributing countries/regions in this field. **(D)** Collaboration network map of countries/regions generated by online analysis platform. **(E)** Overlay visualization map depicting multinational co-authorship analysis generated by using VOSviewer. The color spectrum represents the Average Appearance Year (AAY) for each country/region.

**FIGURE 5:**
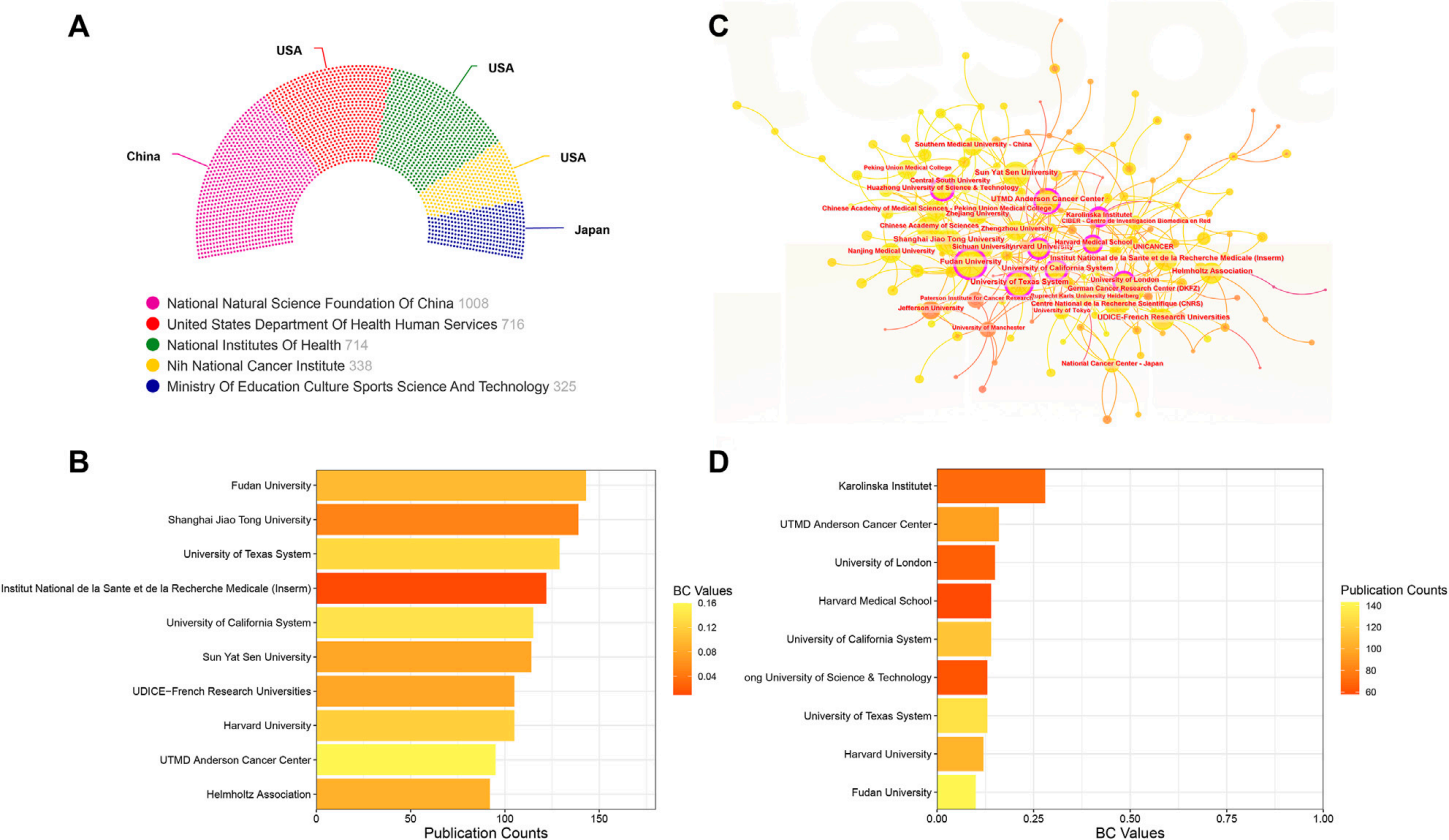
**(A)** The top five funding agencies contributing to this field. **(B)** The top 10 institutions ranked by the number of articles published. **(C)** Collaboration visualization map of institutions generated by CiteSpace. The size of the nodes depends on the number of papers published by the institution. Nodes exhibiting Betweenness Centrality (BC) values exceeding 0.1 are highlighted and encircled in purple. **(D)** The top 10 institutions based on their BC values.

A total of 81 countries/regions have made substantial contributions to research in this domain. Figure 4A showcases the leading 10 contributors based on their publication outputs. The findings highlight China and the United States as the foremost contributors, accounting for 1,839 and 1,300 papers, respectively. Notably, articles from the United States also boast the highest H-index (142) and total citations (85501). In terms of average citations per article, UK leads with an impressive 74.41, closely followed by the United States (65.77) and Italy (57.52) (Figure 4B). Figure 4C displays the annual publication trends of the top 10 contributing countries/regions in this field. As can be seen, China’s annual publication volume surpassed that of the United States in 2019, and has since become the country with the largest annual publication volume. Figures 4D, E offer visual representations of international collaborations among various countries/regions. The connecting lines between them signify collaborative efforts, with line thickness indicating the strength of collaboration. It is evident that the United States engages most intensively in collaborations with China and United Kingdom. Furthermore, in Figure 4E, generated by VOSviewer, countries/regions are color-coded based on their Average Appearance Year (AAY). Blue designations signify early engagement in this field, while red ones denote relatively recent involvement. Additionally, we have compiled information on the top 5 funding agencies contributing to this field in Figure 5A.

#### Contributions of institutions

As for institutions, as illustrated in Figure 5C’s collaboration visualization map generated by CiteSpace, the size of each node corresponds to the volume of publications. Larger nodes indicate more extensive publication records. Additionally, nodes adorned with purple outer rings signify institutions possessing significant centrality, identified by a Betweenness Centrality (BC) value exceeding 0.1. Figure 5B displays the top 10 institutions ranked by the number of articles published. Leading the list is Fudan University, followed closely by Shanghai Jiao Tong University and University of Texas System. Turning to Figure 5D, we summarize the top 10 institutions based on their BC values. Notably, Karolinska Institutet (BC = 0.28), UTMD Anderson Cancer Center (BC = 0.16), and University of London (BC = 0.15) emerge as the three institutions with the highest centrality values.

#### Contributions of authors

**FIGURE 6:**
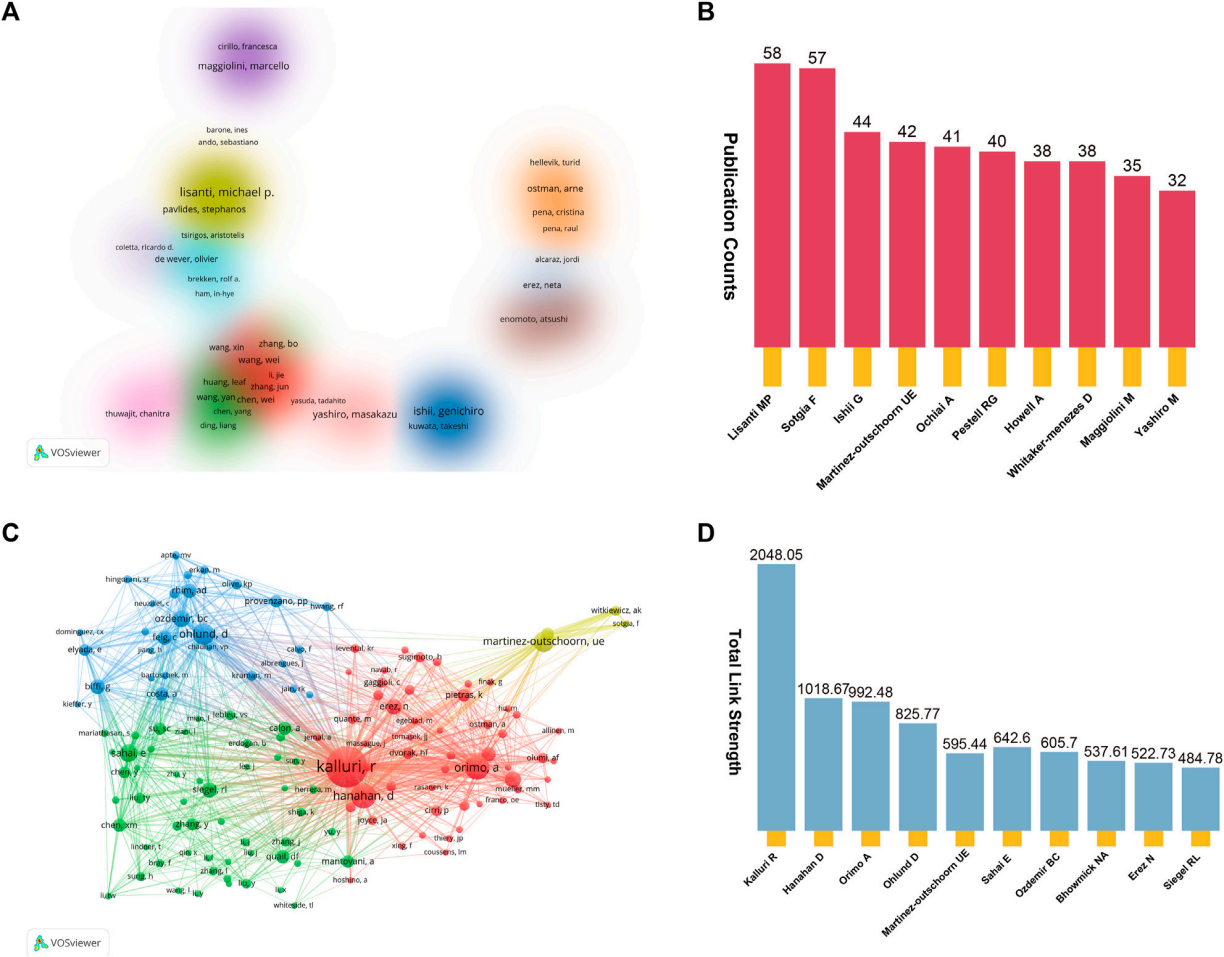
**(A)** Cluster visualization co-authorship analysis of authors with more than eight publications. Altogether, we have pinpointed 16 unique research clusters, each distinguished by a consistent color on the visualization map. **(B)** The top 10 productive authors in this domain. **(C)** Co-cited author relationship network map. **(D)** The top 10 authors with the highest total link strength (TLS).

After conducting a preliminary analysis, it was determined that over 25000 authors actively participated in the 5,190 studies. To streamline the co-authorship cluster visualization in Figure 6A, we focused on authors who had contributed to more than eight publications. Authors sharing the same color-coding within the diagram share similar characteristics in the realm of co-authorship research. These authors were categorized into 16 distinct research clusters. Among these authors, the top 10 individuals who have displayed exceptional productivity through their article publications may prove to be valuable research collaborators and influential figures within the field. As depicted in Figure 6B, Lisanti MP emerged as the most prolific author, closely followed by Sotgia F and Ishii G. Furthermore, the network map showed in Figure 6C illustrates the co-cited author relationship network, where each node represents a cited author, with the node size proportional to the frequency of citations received by the respective author. In Figure 6D, we present a summary of the top 10 authors based on Total Link Strength (TLS), with Kalluri R, Hanahan D, and Orimo A occupying the first three positions.

### Prominently cited research papers

**FIGURE 7:**
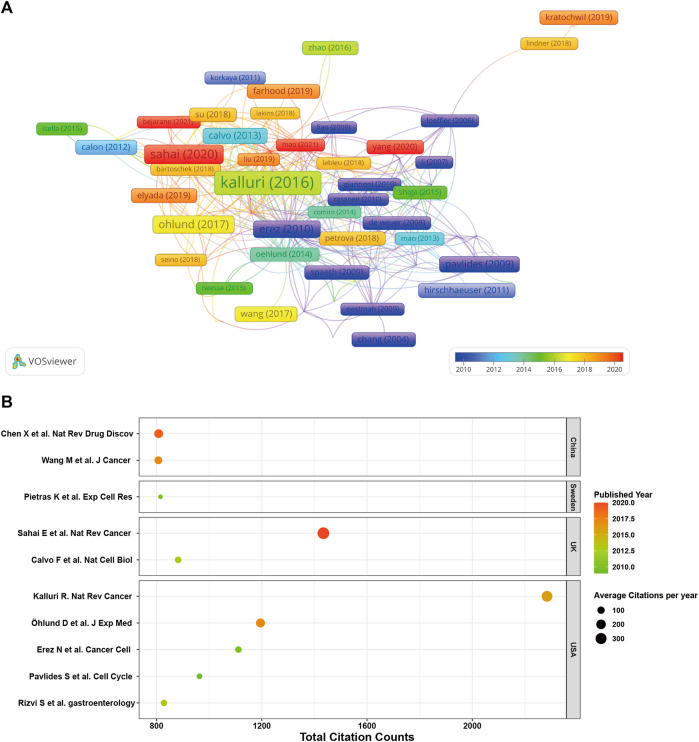
**(A)** Network visualization map of paper citation analysis. **(B)** Detailed insights into the top 10 highly cited studies within the CAFs research domain.

In Figure 7A, we present a network visualization map of paper citation analysis. Larger nodes in the diagram typically represent papers with significantly higher citation counts, indicating their substantial impact and influence within the citation network. Figure 7B offers detailed insights into the top 10 highly cited studies within the CAFs research domain. Remarkably, all these articles were published after 2009. Among them, four papers have accumulated over 1,000 citations each, and all the top 10 have been cited more than 800 times. The paper authored by Kalluri et al. published in 2016 in *Nature Reviews Cancer* claims the highest citation count, amassing an impressive 2,284 citations. Following in the second position is the study by Sahai et al. from 2020, with 1,434 citations, while in the third position stands the paper authored by Öhlund et al., in 2017, having garnered 1,195 citations.

### Visual analysis of reference co-citation and bursting references

**FIGURE 8:**
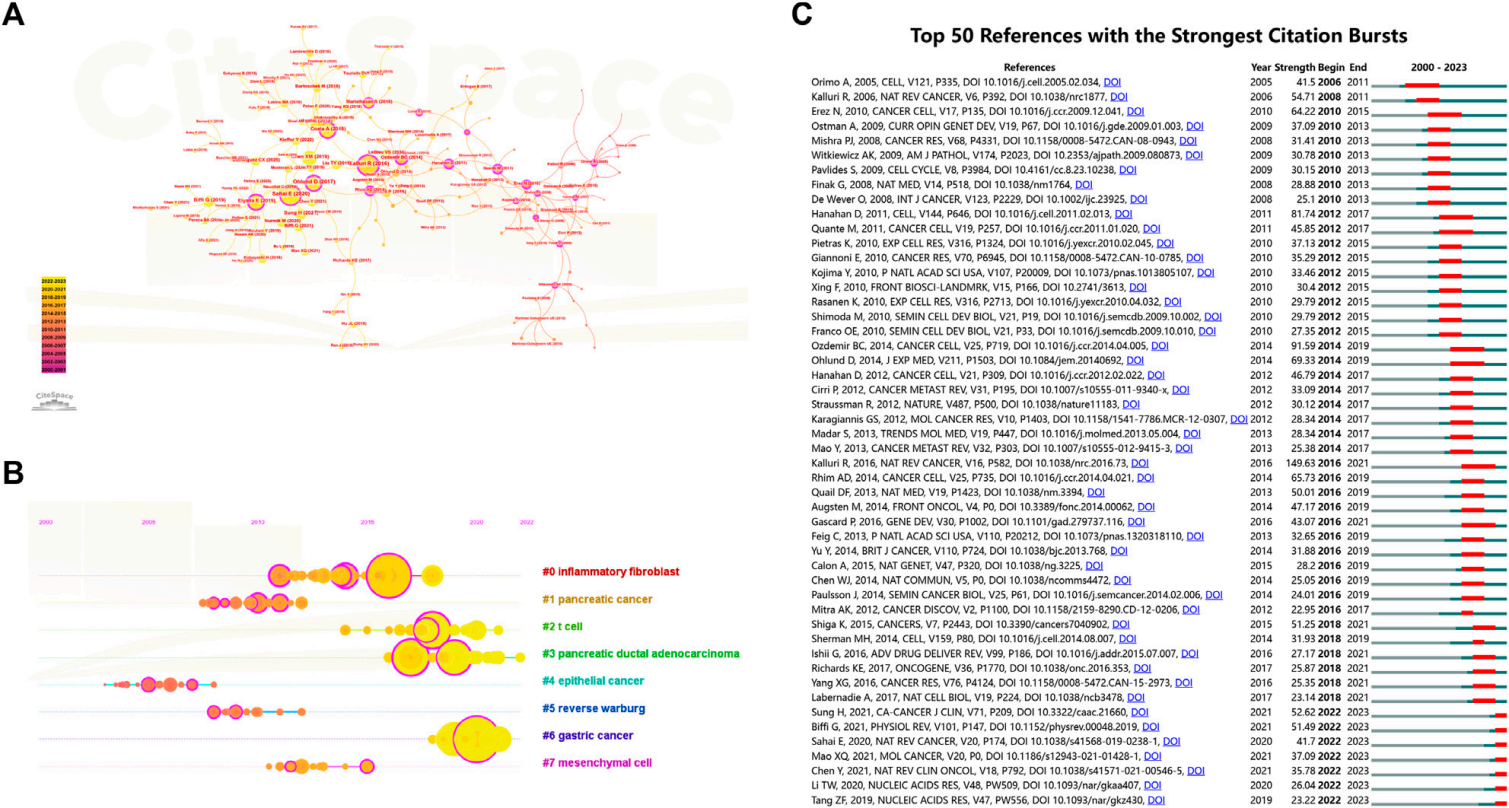
**(A)** Network view map and **(B)** timeline view map of reference co-citation analysis. The application of the clustering function led to the segmentation of the network map into discrete clusters. References within each cluster share similar research directions, distinguishing them from references in other clusters. **(C)** The top 50 references characterized by the most pronounced citation bursts. Blue line segments represent time intervals, whereas the red line segments signify periods of frequent citations.

Reference co-citation analysis, a valuable feature of CiteSpace, is commonly employed to discern prevailing research themes within a specific field. In Figures 8A, B, we present the network view map and timeline view map of reference co-citation analysis. Intra-cluster nodes denote a shared similarity in research focus, and each cluster is denoted by a “#” label, which is extracted from the literature. Additionally, utilizing the log-likelihood ratio algorithm (LLR), we divided all co-cited references into eight distinct clusters. Clusters with Q values exceeding 0.3 and S values surpassing 0.7 signify reliable and significant outcomes. In Figure 8B, the calculated mean S value stands at 0.9332, and the mean Q value is 0.778, underscoring the soundness of the clustering approach. Additionally, this map offers a chronological perspective on these clusters through a timeline view. By utilizing the time axis or the clusters’ average publication year, this timeline view facilitates a rapid evaluation of each cluster’s evolutionary dynamics. Notably, the prevailing research focus has transitioned towards gastric cancer, T cell, and pancreatic ductal adenocarcinoma. References exhibiting citation bursts signify that several studies have garnered noteworthy attention from the scholarly community during distinct timeframes. As illustrated in Figure 8C, we have identified the top 50 references characterized by the most pronounced citation bursts.

### Visual analysis of keywords co-occurrence and bursting keywords

**FIGURE 9:**
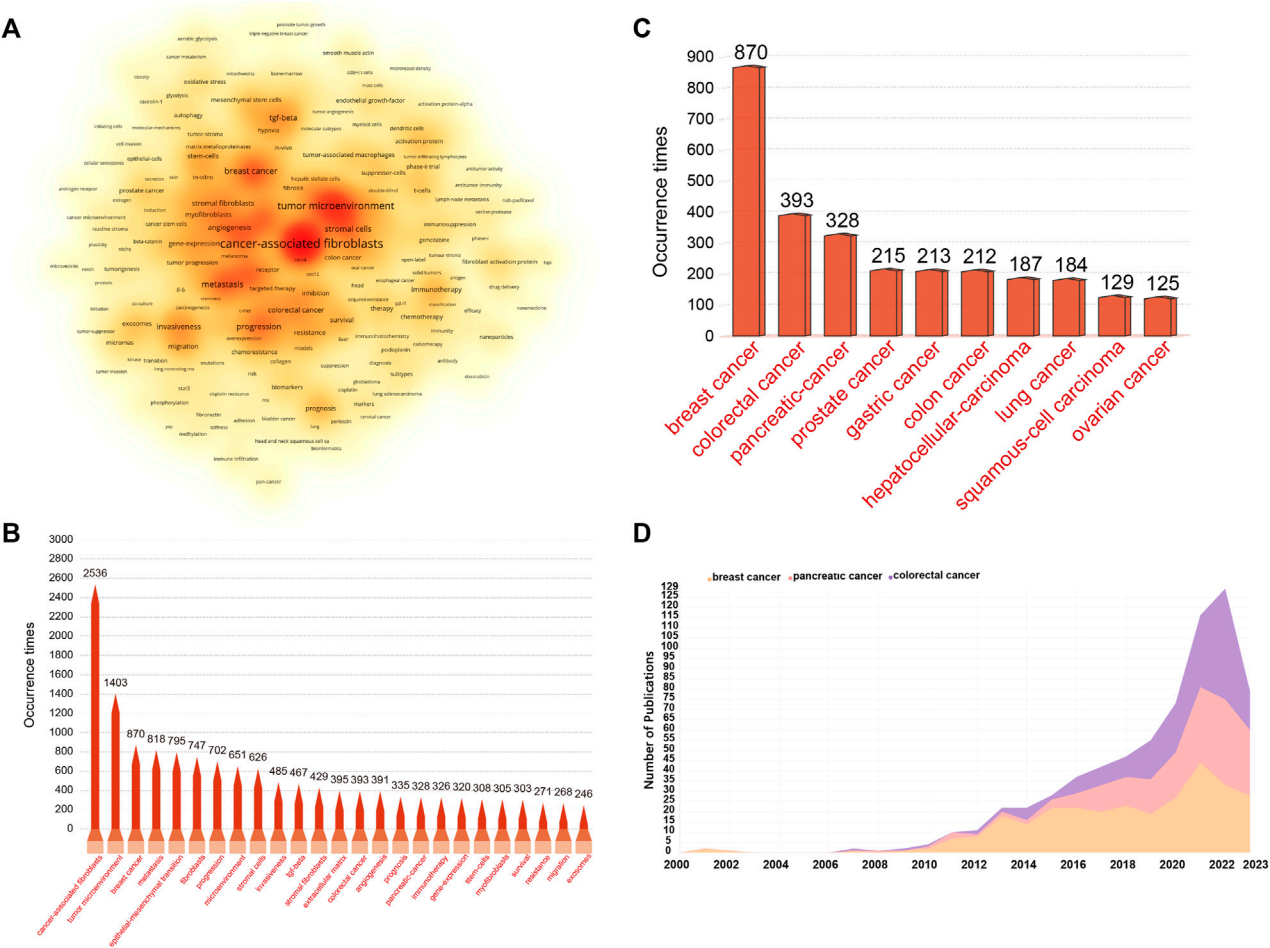
**(A)** Density visualization map depicting the analysis of keyword co-occurrence. This heat map visually portrays keyword frequency using a spectrum of color shades. Deep red shades signify vibrant research areas with a higher frequency of keyword co-occurrence, while milder yellow shades represent less active areas with a lower frequency of keyword co-occurrence. **(B)** Frequency distribution of the top 25 most frequently studied keywords. **(C)** The top10 most studied tumors in the field of CAFs. **(D)** The annual publication trend of the top 3 most studied tumors.

**FIGURE 10:**
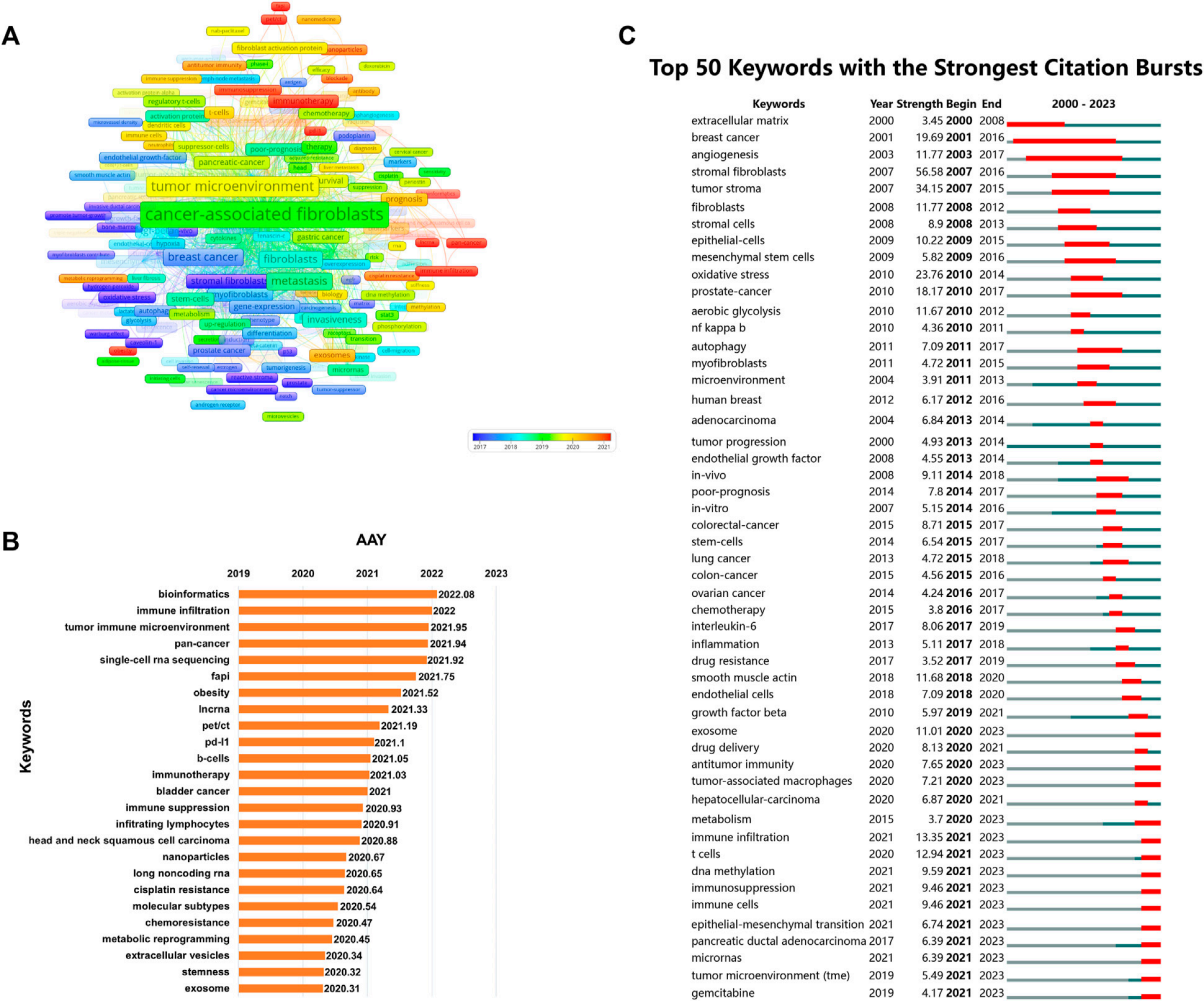
**(A)** Overlay visualization map of co-occurrence keywords. **(B)** The top 25 keywords with the highest AAY. **(C)** The top 50 keywords characterized by the most pronounced citation bursts.

Keywords with high frequencies could act as valuable indicators of current research areas of interest. From a compilation of 5,190 publications, we extracted a total of 292 keywords that appeared more than 20 times. Figure 9A illustrates the density co-occurrence map of these prominent keywords. Figure 9B presents the frequency distribution of the top 25 most frequently studied keywords. It is evident that, in addition to the search terms of CAFs, other prevalent keywords in the research domain include tumor microenvironment, breast cancer, metastasis, epithelial-mesenchymal transition, and so on. In addition, we also summarized the top10 tumors studied in the field of CAFs. As can be seen from Figure 9C, breast cancer was the most studied tumor, followed by colorectal cancer, pancreatic-cancer, prostate cancer, and gastric cancer. By using the online website, we analyzed the annual publication trend of the top three tumors including breast cancer, colorectal cancer, and pancreatic-cancer in Figure 9D. Furthermore, we also provide the overlay visualization map of these keywords in Figure 10A. In this map, these keywords were color-coded according to their AAY, with keywords that appeared earlier marked in blue and those with a more recent appearance marked in red. Figure 10B displayed the top 25 keywords including bioinformatics, immune infiltration, tumor immune microenvironment, etc., with the highest AAY. Moreover, CiteSpace was also utilized to identify emerging research trends and cutting-edge topics through the analysis of outbreak terminologies. As illustrated in Figure 10C, we identified a set of 50 keywords displaying significant citation bursts. Remarkable keywords characterized by ongoing burst included exosome (2020–2023), antitumor immunity (2020–2023), tumor-associated macrophages (2020–2023), metabolism (2020–2023), immune infiltration (2021–2023), T cells (2021–2023), DNA methylation (2021–2023), immunosuppression (2021–2023), immune cells (2021–2023), epithelial-mesenchymal transition (2021–2023), pancreatic ductal adenocarcinoma (2021–2023), microRNAs (2021–2023), tumor microenvironment (2021–2023), and gemcitabine (2021–2023).

### Exploration of key genes in focus

**FIGURE 11:**
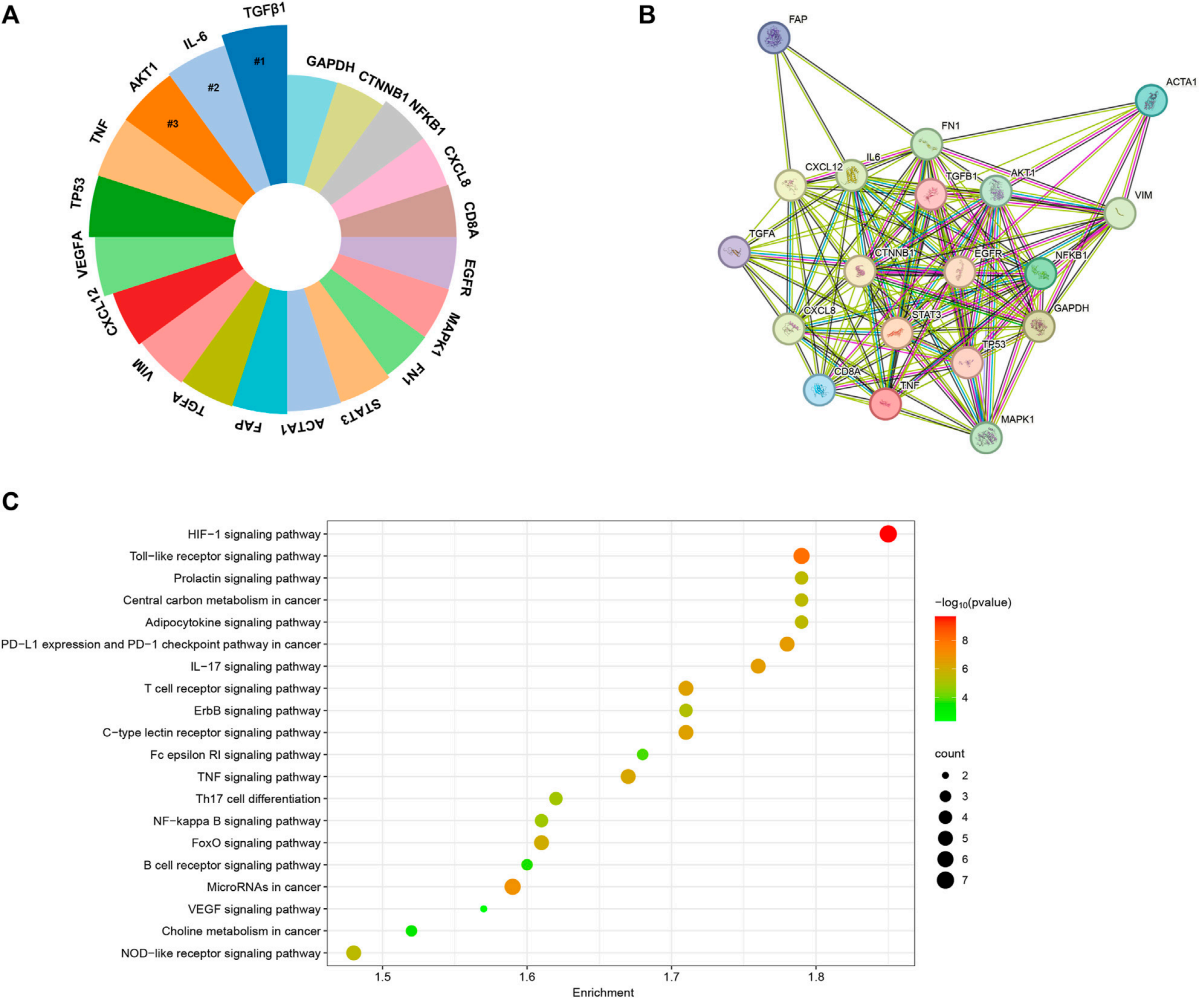
**(A)** The top 20 genes garnered the greatest research attention in the field of CAFs. **(B)** The protein-protein interaction network generated utilizing the STRING tool. **(C)** KEGG enrichment analysis of the top 20 genes.

We conducted a comprehensive analysis of the most extensively studied genes within the realm of CAFs by using an online data analysis platform. As illustrated in Figure 11A, TGFB1, IL-6, TNF, TP53, and VEGFA emerged as the top five genes that have garnered the greatest research attention in the field of CAFs. To delve deeper into the potential molecular mechanisms of these genes in the wound healing process, we employed the STRING tool to acquire a protein-interaction network (Figure 11B). Furthermore, we conducted KEGG enrichment analyses on these top 20 relevant genes, presenting the results in bubble plots. As depicted in Figure 11C, the KEGG enrichment analysis highlighted that these genes were mainly associated with HIF-1and Toll-like receptor signaling pathways.

## Discussion

### General information

In the field of scientific research, particularly within rapidly evolving domains, gaining insight into current research trends and frontiers has become paramount. The era of big data has ushered in a vast volume of scientific literature and information, making it increasingly challenging for researchers to track and analyze the latest research developments. However, bibliometric analysis, through quantitative examination of literature data, provides a valuable means to enhance our understanding of research progress, identify research hotspots, and forecast future research directions (Shen et al., 2022a; Cheng et al., 2022). To our knowledge, this is the first bibliometric report in the field of CAFs. In this study, a total of 5,190 documents including 4,070 articles and 1,120 reviews were ultimately incorporated. In comparison to other bibliometric studies that summarizing the global research trends in tumor-related cells such as tumor-associated macrophages, that included 6,405 articles between 2001 and 2021, CAFs deserves further attention (Zhou et al., 2023). Regarding the annual volume of publications, the period from 2000 to 2006 could be considered as the first phase, characterized by a stable trend with almost no growth, averaging four publications per year. The period from 2007 to now could be viewed as the second phase, during which high-quality research in this field were progressively published worldwide. The fitting curves showed the exponential growth in the second stage. In addition, according to our statistics, all the top 10 highly cited studies within the CAFs research domain were published after 2009 (Pavlides et al., 2009; Erez et al., 2010; Calvo et al., 2013; Kalluri, 2016; Öhlund et al., 2017; Sahai et al., 2020).

As for the major contributors in this field, it is evident that China and the United States play dominant roles, accounting for more than half of all publications. Additionally, the United States boasts the highest H-index and total citations, establishing itself as the primary influencer in this research domain, both in terms of quantity and quality. As we all know that financial support stands as a primary driving force behind scientific research. Among the top five funding agencies contributing to this field, China and the United States clearly providing the largest amount of funded publications. In addition, it is worth noting that certain countries, despite having a smaller number of published papers, have achieved remarkably high average citations per article. UK leads with an impressive 74.41, closely followed by the United States (65.77) and Italy (57.52). This elevated average citation rate could be attributed to the publication of several highly influential studies. For instance, among the top 10 highly cited studies, at least the first author of two studies came from UK (Calvo et al., 2013; Sahai et al., 2020). Among them, one consensus statement aimed to consolidate the existing knowledge and provide a roadmap for furthering comprehension of this pivotal cell population within the tumor microenvironment has acquired more than 1,400 citation times (Sahai et al., 2020). Turning our attention to institutional analysis, the top three institutions in terms of publication volume were the Fudan University, Shanghai Jiao Tong University and University of Texas System. However, it is worth mentioning that the level of inter-institutional collaboration has been relatively low, primarily limited to countries/regions. Recognizing this, fostering collaborative networks among diverse research institutions or teams will be of paramount importance for future studies.

### Research hot spots and frontiers

Bibliometrics employs techniques such as co-citation analysis of references, word frequency and co-occurrence analysis of keywords, and burst word analysis to discern the research direction, prevailing research topics, and cutting-edge areas within a specific field (Shen et al., 2022b; Liu et al., 2022). Combining the results from co-occurrence and bursting keywords, we identified the following research topics including immune cells (T cells, B-cells, tumor-associated macrophages), tumor immune microenvironment (antitumor immunity, immune infiltration, immunosuppression), immunotherapy (PD-L1), microRNAs (miRNA), extracellular vesicles (exosome), multiple tumors (pancreatic ductal adenocarcinoma, bladder cancer, head and neck squamous cell carcinoma), antitumor agents (gemcitabine, cisplatin resistance), bioinformatics (pan-cancer), epithelial-mesenchymal transition (stemness), FAPI PET/CT, DNA methylation, etc., may receive sustained attention in the future. As a matter of fact, several outstanding review articles detailing exosome or microRNAs mediating crosstalk between CAFs and cancer cells, or the role of CAFs in many tumors, have been thoroughly documented, and interested readers are encouraged to consult them for further information (Pavlides et al., 2009; Erez et al., 2010; Calvo et al., 2013; Kalluri, 2016; Sahai et al., 2020). Due to space limitations and a vast amount of available reviews, we only take immunotherapy, FAPI PET/CT, and DNA methylation as examples to talk their relationships with CAFs.

#### CAFs and immunotherapy

While immunotherapy exhibits superior effectiveness and tolerability compared to conventional and targeted therapies, numerous patients still exhibit intrinsic or acquired resistance. This issue is particularly prominent in the case of advanced solid tumors, where inhibitor therapies targeting Programmed Death Receptor 1 (PD-1) and Programmed Death Ligand 1 (PD-L1) have become central. As a prominent constituent of the tumor microenvironment, CAFs engage in interactions with cancer cells and immune cells through the secretion of cytokines and vesicles, involvement in extracellular matrix remodeling, ultimately influencing the immune response process (Li et al., 2018; Li et al., 2019; Dou et al., 2020). Previous studies revealed that in melanoma and colorectal carcinoma, CAFs could release C-X-C motif chemokine ligand 5 (CXCL5), which binds to the C-X-C motif chemokine receptor 2 (CXCR2) on cancer cells (Li et al., 2019). This interaction subsequently activates the PI3K/AKT signaling pathway, leading to an increase in PD-L1 expression on the cancer cell surface, ultimately culminating in immune evasion. In addition, another study found that CAF-derived exosomes microRNA-92 (miR-92) induced the expression of PD-L1 in breast cancer and subsequently leading to the apoptosis of T cells and impairment of NK cell function (Dou et al., 2020). Mechanically, this was associated with the downregulation of miR-92 target gene LATS2 and enhancement of the nuclear translocation of YAP1, which further enhanced PD-L1 transcription activity. Beyond their role in modulating the expression of PD-L1 on the surface of cancer cells, CAFs also exert an impact on immune efficacy through the regulation of immune cell differentiation and extracellular matrix remodeling. For example, CAFs undergo glycolysis, resulting in the release of significant amounts of lactate and hydrogen ions, thus creating an acidic microenvironment that inhibits immune cell activity (Pavlides et al., 2009). Meanwhile, CAFs have the capacity to modify the extracellular matrix through the secretion of collagen and fibronectin, resulting in increased tumor stiffness (Lu et al., 2023). This alteration hinders the movement of T cells, particularly CD8^+^ T cells, creating a barrier effect against the infiltration of drugs and immune cells and ultimately leading to the suppression of anti-tumor immunity (García-Palmero et al., 2016; Zhang et al., 2016). In light of the immunosuppressive effects employed by CAFs in resistance to PD-1/PD-L1 immunotherapy, therapeutic interventions targeting key molecules within the underlying mechanism such as TGF-β, NOX4 and exosome, are being explored in clinical trials to augment tumor immune efficacy against PD-1/PD-L1 (Pan et al., 2020; Kato et al., 2021; Li et al., 2023). Nonetheless, recent investigations indicated the presence of distinct CAFs subpopulations including tumor-suppressing and tumor-promoting phenotypes with differing molecular profiles and functions (Öhlund et al., 2017). Hence, to enhance the precision of combined targeted therapy, the identification of more reliable and specific anti-tumor CAFs biomarkers will be imperative in the future.

#### CAFs and FAPI PET/CT

Although 18F-fluorodeoxyglucose (18F-FDG) positron emission tomography (PET) has revolutionized the field of tumor imaging, its specificity and sensitivity are limited in certain tumor subtypes (Chandekar et al., 2023). Fibroblast activation protein (FAP) is a type II integral membrane glycoprotein expressed by CAFs and has been identified as a crucial biomarker for CAFs (Rezaei et al., 2023). Importantly, FAP is involved in various pathological processes and is selectively overexpressed in majority of tumors (over 90% of malignant epithelial tumors), making it a potential pan-cancer target (Chen and Song, 2019; Fitzgerald and Weiner, 2020). Researchers have developed various FAP-targeted probes for imaging different tumors, ranging from antibodies to boron-based inhibitor molecules, and have identified quinoline-based FAP inhibitors (FAPI) as candidates for radiopharmaceuticals in FAPI PET/CT imaging (Lindner et al., 2018). Preliminary preclinical data from FAPI PET/CT is promising, and extensive multidisciplinary clinical studies are currently underway for the diagnosis and treatment of various malignant tumors and their metastases (Alçın et al., 2023; Zhang et al., 2023b; Demmert et al., 2023). In recent years, the utility and effectiveness of FAPI PET/CT in tumor detection and staging have been compared with FDG PET/CT in various clinical settings, including standardized uptake values, uptake rates, and clearance rates (Qiao et al., 2023). In various clinical contexts, FAPI imaging has been shown to outperform 18F-FDG (Alçın et al., 2023; Zhang et al., 2023b; Qiao et al., 2023). Although widespread adoption in clinical practice is not yet realized, it can be inferred that FAPI imaging may offer new perspectives in tumor diagnosis and management compared to traditional imaging and radiotherapy approaches.

#### CAFs and DNA methylation

CAFs constitutes the most abundant population in the tumor stroma and promotes tumorigenesis by secreting various factors that regulate intercellular signaling in tumor cells, leading to the remodeling of cancer tissue. Studies have shown that DNA methylation plays a crucial role in mediating the pro-cancer activity of fibroblasts (Halperin et al., 2022; Kehrberg et al., 2023). Changes in the methylation levels of specific genes (either increased or decreased) contribute to the activation of CAFs. It was found by Yu et al. (Yu et al., 2012) that CAFs derived from pancreatic ductal adenocarcinoma displayed lower methylation levels and overexpression of ADAM metallopeptidase domain 12 compared to normal fibroblasts. Vizoso et al. (Vizoso et al., 2015) conducted genome-wide human methylation microarray analysis of CAFs from non-small cell lung cancer patients and paired control fibroblasts. Compared to normal fibroblasts, CAFs exhibited overall DNA hypomethylation along with some hypermethylated genes. These DNA methylation changes selectively influenced gene expression in the TGF-β pathway. Another study found that normal fibroblasts could be reprogrammed into a pro-invasive phenotype by induction with leukemia inducible factor (LIF) (Albrengues et al., 2015). This process was primarily associated with DNA methylation of the LIF-induced protein phosphatase regulator SHP1 (*SHP-1*) promoter and *SHP-1* loss, resulting in constitutive activation of JAK1/STAT3 signaling. Treatment of CAFs with DNA methyltransferase inhibitors restored SHP-1 expression and reduced the invasiveness of CAFs. However, due to the lack of animal models studying the epigenetics of CAFs in the tumor microenvironment, most studies have been conducted *in vitro*. More research is needed to help understand the mechanisms through which DNA methylation or demethylation regulates CAFs behavior in the tumor microenvironment.

### Hot genes in focus

In this study, we utilized an online data analysis platform to compile an extensive list of genes that have been extensively investigated in the context of CAFs. Our findings highlighted the significance of specific genes, including TGFB1, IL-6, AKT, TNF, TP53, VEGFA, CXCL12, VIM, TGFA, FAP, ACTA1, STAT3, FN1, MAPK1, EGFR, CD8A, CXCL8, NFKB1, CTNNB1, and GAPDH. These genes have garnered considerable research attention due to their involvement in the signaling pathway and molecular mechanisms related to CAFs. For example, TGF-β plays a crucial role in the activation and generation of CAFs, contributing to the preservation of CAF morphological traits and functional characteristics (Watabe et al., 2023). Inside the tumor tissue, the three established isoforms of TGFβ, namely, TGFβ1, TGFβ2, and TGFβ3, are released by cancer cells or CAFs (Micke and Ostman, 2005). Recent efforts have been directed toward unraveling the mechanisms through which TGF-β facilitates CAFs formation, yielding a range of intriguing findings (Yao et al., 2018; Pazhani et al., 2022). To delve into the potential molecular mechanisms underlying the roles of these genes in CAFs, we expanded our analysis by constructing a protein-interaction network using the STRING tool. This network allowed us to visualize the intricate interactions among the proteins encoded by these genes and how they might collectively regulate the complex processes associated with CAFs. Furthermore, to gain deeper insights into the biological significance of these top 20 related genes, we conducted KEGG enrichment analyses. The results of this analysis revealed that HIF-1and Toll-like receptor signaling pathways were significantly enriched. The HIF-1 signaling pathway represents a crucial cellular mechanism for responding to low-oxygen environments. It assists cells in adapting to situations with insufficient oxygen supply by modulating gene expression. In the context of cancer, the HIF-1 signaling pathway promotes tumor growth, invasion, and drug resistance through various mechanisms. Numerous previous studies have demonstrated the significant involvement of HIF in the metabolic reprogramming of CAFs, thereby mediating the pro-tumorigenic impact of CAFs (Du et al., 2015; Rohwer et al., 2019). Of note, apart from HIF-1, one recent study also revealed that the deletion of CAFs-HIF-2α could led to a moderate reduction in tumor fibrosis and a significant decrease in the recruitment of immunosuppressive M2 macrophages within the pancreatic ductal adenocarcinoma. Consequently, this deletion resulted in an enhanced response to immunotherapy within the tumor. Their findings propose that stromal HIF-2 represents a critical element in the pathobiology of PDAC and presents a feasible therapeutic target. Targeting stromal HIF-2 holds the potential to alleviate immunosuppression within the tumor microenvironment and boost immune responses against this disease (Garcia Garcia et al., 2022).

### Limitation

Our research exhibits several limitations. Firstly, we exclusively relied on the WoSCC database, neglecting other prominent databases such as Pubmed, Scopus, and Embase. This limitation constrains the comprehensiveness of our study. However, to our knowledge, previous studies mostly used a single database due to differences in data formats among different databases. Of them, WoSCC is generally considered the most appropriate database (Cheng et al., 2023b; Wu et al., 2023). Secondly, even though the WoSCC database is renowned for its high-quality research, our selection was limited to articles and reviews, introducing an inherent selection bias. Thirdly, only literature written in English was included in this study, which may also result in a statistic bias. Additionally, the presence of authors sharing identical initials and variations in reference versions could introduce potential inaccuracies in our analyses. Nevertheless, we believe our data is representative and the results could be generalizable.

## Conclusion

The research on CAFs represents a significant hotspot in the field of cancer research, with their roles and regulatory mechanisms continuously being unveiled and further elucidated. In-depth investigations into CAFs hold the potential to usher in novel breakthroughs and strategies in cancer therapy. This study represents the first bibliometric analysis conducted on the trends in CAFs research spanning the last 2 decades. The findings of this analysis hold potential for researchers to discern novel avenues of investigation and gain a comprehensive understanding of prevailing research patterns and emerging focal points within this domain. We suggest that the field of CAFs represents an area of research that remains inadequately explored, especially the role in the immune microenvironment and the interaction with immune cells, necessitating further investigation to acquire robust scientific evidence for better cancer therapy. At the same time, our data could provide valuable reference for scholars, especially young researchers, to carry out similar research in this field. Meanwhile, it is anticipated that enhanced collaboration among authors, institutions, and nations will facilitate future advancements in this field.

## Data Availability

The original contributions presented in the study are included in the article/supplementary material, further inquiries can be directed to the corresponding authors.
